# Unscheduled m^6^A Deposition in RNA via m^6^ATP Incorporation by DNA Polymerases

**DOI:** 10.3390/ijms26199263

**Published:** 2025-09-23

**Authors:** Fei Qu, Jeanpierre Fuente, Prem Chapagain, Yuan Liu

**Affiliations:** 1 Biochemistry Ph.D. Program, Florida International University, Miami, FL 33199, USA; fqu002@fiu.edu (F.Q.); jfuen076@fiu.edu (J.F.); chapagap@fiu.edu (P.C.); 2 Department of Physics, Florida International University, Miami, FL 33199, USA; 3 Biomolecular Sciences Institute, Florida International University, Miami, FL 33199, USA; 4 Department of Chemistry and Biochemistry, Florida International University, Miami, FL 33199, USA

**Keywords:** N^6^-methyladenosine (m^6^A), unconventional m^6^A deposition on RNA, m^6^ATP incorporation by DNA polymerases, DNA polymerase η, DNA polymerase β, AlphaFold3-assisted molecular dynamics simulation

## Abstract

N^6^-methyladenosine (m^6^A) is the most abundant modification of mRNA and plays a crucial role in mediating cellular functions, and it is associated with cancer and neurodegenerative diseases. Studies have shown that m^6^A is predominantly deposited on its consensus motif by the m^6^A writer proteins RNA methyltransferase METLL3/METLL14. However, it was found that nonconventional m^6^A deposition by other alternative pathways may also exist and can modulate epitranscriptomic regulation in cells. Thus, understanding the molecular mechanisms underlying nonconventional m^6^A deposition outside the canonical motifs will provide novel insights into the full scope of the functional impact of m^6^A. In this study, we discovered that m^6^ATP was efficiently incorporated by the repairing DNA polymerases pol β and pol η through RNA gap-filling synthesis on an RNA-DNA hybrid. Steady-state kinetics results showed that m^6^ATP was incorporated into RNA by the DNA polymerases with a comparable efficiency to ATP. AlphaFold3-assisted molecular dynamics simulations further elucidated the structural basis for the DNA polymerases to incorporate m^6^ATP into the RNA substrates by showing that the enzymes employed the unique base-stacking mechanism to govern the distance between the 3′-OH group of the 3′-terminus nucleotide of the primer and the 5′-α-phosphate of m^6^ATP to perform their catalysis. Furthermore, we detected a significant amount of m^6^ATP in human cells. We showed that the m^6^ATP level was associated with that of the oxidative stress biomarker 8-oxoGTP in cells, suggesting that unscheduled m^6^A deposition on RNA can be mediated by m^6^ATP incorporation that is associated with cellular oxidative stress. Our study sheds light on the unscheduled m^6^A deposition as a potential alternative mechanism for altering epitranscriptomic modifications.

## 1. Introduction

m^6^A is the most abundant internal modification identified in the eukaryotic mRNA [1]. m^6^A plays a central role in regulating gene expression by modulating RNA alternative splicing [2], stability [3,4], nuclear export [5], and protein translation [6]. Thus, as a critical epitranscriptional marker, m^6^A is involved in the regulation of cellular stress responses [7,8], disease status [9,10,11], and cancer development [12,13]. It has been shown that m^6^A exists in all the regions of mRNA but preferentially accumulates around the translation start site and the stop codons [14]. m^6^A-seq has revealed that most m^6^A is found within the consensus m^6^A motif, R/DRACH (1), in which R/D represents G or A, and H represents U or C or A. However, studies have shown that the m^6^A motifs are degenerate, with multiple variations occurring at the +/− 1, 2, and 3 positions [1,14,15,16]. Moreover, it was shown that m^6^ATP can also be deposited by another m^6^A writer, METTL16, at a different motif of UACAGAGAA [17]. All these findings suggest that there are diversified alternative pathways for noncanonical m^6^A deposition on RNA through the pathways mediated by known or unknown m^6^A methyltransferases, as well as non-methyltransferase-dependent mechanisms. It was shown that the locations of m^6^A play an essential role in regulating cellular functions. For example, m^6^A in the 5′-UTR can facilitate cap-independent translation by recruiting a specific m^6^A reader, eukaryotic initiation factor 3 (eIF3) [18], whereas m^6^A located around the stop codons and in the 3′-UTR is recognized by the m^6^A reader, YTHDF2, mediating RNA decay [3]. While m^6^A deposition by the RNA methyltransferases and m^6^A writer METTL3/METTL14 exhibits a preference for the R/DRACH motifs [19], the presence of m^6^A in noncanonical sites in RNA raises intriguing questions about whether there are alternative mechanisms for m^6^A RNA deposition. Understanding its underlying mechanisms is crucial for elucidating the entire regulatory role of m^6^A RNA modifications in cellular processes.

Previous studies have revealed that wild-type and mutant DNA polymerases can incorporate ribonucleoside triphosphates (rNTPs) into DNA through their compromised ability to exclude rNTPs [20]. Gao et al. have further demonstrated that the mutant MMLV reverse transcriptase can acquire RNA polymerase activity to incorporate rNTPs into RNA [21]. Recent studies have shown that pol η, a translesion synthesis DNA polymerase [22], which is essential in assisting replication DNA polymerases to bypass bulky DNA lesions during DNA replication [23], also exhibits RNA synthesis activity [24]. This unexpected capability of DNA polymerases to incorporate ribonucleotides suggests a broader spectrum of substrate recognition of repairing DNA polymerases and their functional flexibility and plasticity in processing both deoxyribonucleotides and ribonucleotides, mediating DNA repair, and potentially processing RNA damage intermediates, especially on RNA-DNA hybrids formed during gene transcription and DNA lagging-strand replication. Furthermore, it was found that RNA synthesis activity by pol η can be significantly increased by manganese (Mn^2+^) compared with its RNA synthesis activity in the presence of Mg^2+^ [25,26]. This further raises the question of whether repairing DNA polymerases can also incorporate m^6^ATP, the methylated ATP, into RNA through their RNA synthesis activity on DNA-RNA hybrids.

Although both the divalent metals Mg^2+^ and Mn^2+^ are essential cofactors for the catalysis of nucleotide incorporation of DNA polymerases [27], Mg^2+^ is the physiologically relevant metal cofactor for DNA polymerases [28,29]. This is because Mg^2+^ is abundant in cells and can provide the optimal conditions for efficient enzymatic activity of DNA polymerases and the high fidelity of DNA synthesis [30]. On the other hand, although Mn^2+^ is an essential trace metal in cells [28,29], at the level where it can significantly increase DNA polymerase activities, Mn^2+^ can also substantially reduce the stringency of nucleotide selection and fidelity of DNA polymerases, thereby promoting the misincorporation of nucleotides and mismatches [31]. This further suggests that Mn^2+^ can modulate the structures of DNA polymerases, making the enzymes more adaptable to various types of modifications of nucleotides, such as different configurations of ribose and base modifications. However, it is unknown if Mn^2+^ can cause pol η to incorporate the modified ribonucleotide m^6^ATP and if the metal can also induce the structural changes of the high-fidelity DNA repair polymerase, DNA polymerase β (pol β), the central component of the DNA base excision repair (BER) pathway [32,33], which also possesses deoxyribose-5-phosphate (5′-dRP) lyase activity [33], conferring pol β RNA synthesis activity and its incorporation of modified ribonucleotides such as m^6^ATP. Understanding the incorporation of m^6^ATP by the repairing DNA polymerases and the underlying mechanism can open a new avenue to reveal an alternative mechanism for m^6^A RNA deposition, in addition to the canonical m^6^A deposition at the R/DRACH motif by RNA methyltransferases. Therefore, it is crucial for deciphering the potential mechanisms of m^6^A deposition beyond its canonical methylation by the writer enzymes. We further hypothesized that repairing DNA polymerases such as pol β and pol η can incorporate m^6^ATP in the presence of Mg^2+^ and Mn^2+^. To test this hypothesis, we initially determined the activity of pol β and pol η in incorporating ATP and m^6^ATP into RNA using Mg^2+^ or Mn^2+^ as a cofactor. We then compared the efficiency of m^6^ATP incorporation with that of ATP by pol β and pol η using steady-state enzyme kinetics. We further revealed the structural basis of the incorporation of m^6^ATP by pol β and pol η with Mg^2+^ and Mn^2+^ using molecular modeling and molecular dynamics (MD) simulations. Finally, we determined the level of m^6^ATP in human cells. Our results showed that pol β only incorporated m^6^ATP in the presence of Mn^2+^ with high efficiency, whereas pol η efficiently incorporated m^6^ATP in the presence of both Mg^2+^ and Mn^2+^.

## 2. Results

### 2.1. Pol β Can Incorporate m^6^ATP into RNA in the Presence of Mn^2+^

**Figure 1:**
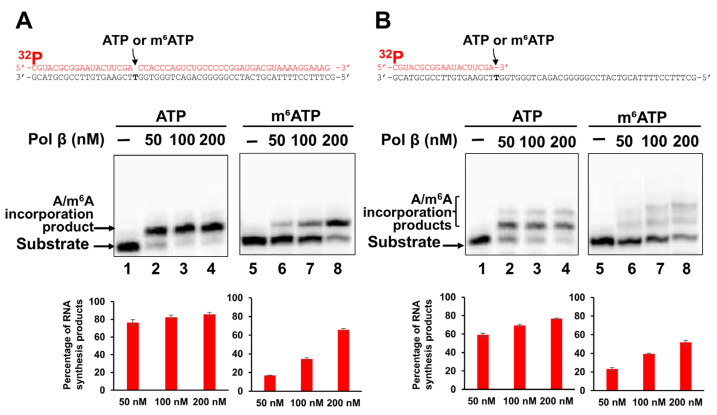
**Incorporation of ATP and m^6^ATP into RNA by pol β in the presence of Mn^2^**^+^. DNA-templated 1 nt RNA gap and open-template RNA substrates (50 nM) were incubated with increasing concentrations of pol β (50, 100, and 200 nM) and 500 μM ATP or m^6^ATP at 37 °C for 60 min in the presence of 1 mM Mn^2+^. Panel (**A**) represents the pol β RNA synthesis resulting from a 1 nt RNA gap substrate and the quantification of the RNA synthesis products. Panel (**B**) represents the pol β RNA synthesis products on the open RNA template substrate. Lanes 1 and 5 represent substrate only. Lanes 2–4 represent pol β ATP incorporation on the gap or open-templated RNA substrate. Lanes 6–8 represent pol β m^6^ATP incorporation on the gap or open-templated RNA substrate. The synthesized RNA products are indicated by arrows on the left side of the gel images. The percentage of the pol β RNA synthesis products was calculated and illustrated in the bar charts below the gel images. All experiments were performed at least in triplicate.

Since previous studies have shown that pol η can synthesize RNA [24,25,26], we further hypothesized that RNA gaps resulting from oxidative RNA damage on RNA-DNA hybrids can allow the incorporation of m^6^ATP into RNA by the repairing DNA polymerases. To test this possibility, we initially determined pol β m^6^ATP and ATP incorporation into the 1 nt RNA gap and open-template substrates in the presence of 5 mM Mg^2+^. We found that pol β failed to incorporate either ATP or m^6^ATP on the gapped or open-template RNA in the presence of Mg^2+^ s. Since the previously reported structures of pol β with Mg^2+^ or Mn^2+^ have revealed that pol β adopts a more open structure in the presence of Mn^2+^ [34], we then tested whether pol β can incorporate m^6^ATP into RNA in the presence of Mn^2+^. The results showed that pol β incorporated m^6^ATP in both gapped and open-template RNA in the presence of 1 mM Mn^2+^ (Figure 1). Pol β at 50–200 nM inserted only one ATP or m^6^ATP into the 1 nt RNA gap substrate, with a much higher amount of ATP incorporation product (Figure 1A, lanes 2–4) than that of m^6^ATP (Figure 1A, lanes 6–8). On the open RNA template substrate, the same concentrations of pol β inserted two ATP or m^6^ATP (Figure 1B), with much more ATP incorporation products generated (Figure 1B lanes 2–4) than m^6^ATP incorporation products (Figure 1B, lanes 6–8). This indicated that m^6^A was extended by additional m^6^A incorporation in the presence of Mn^2+^. The results showed that m^6^ATP could be incorporated by pol β only in the presence of Mn^2+^, with a lower efficiency compared with its ATP incorporation. Moreover, we found that the extension of m^6^A by pol β resulted in m^6^A:G mismatch.

### 2.2. Incorporation of m^6^ATP into RNA by Pol η

**Figure 2:**
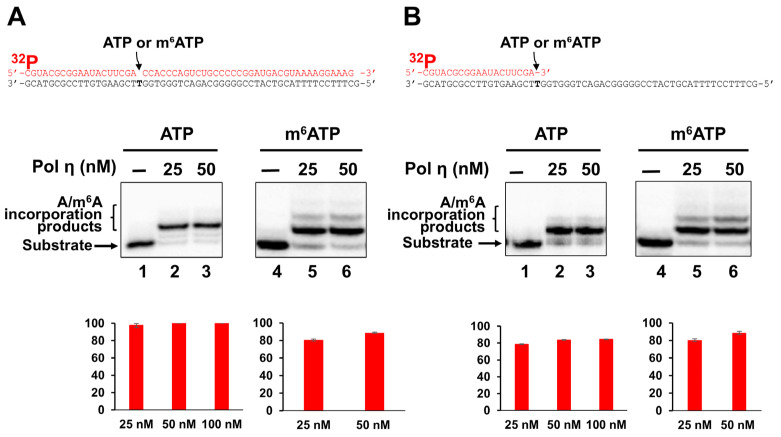
**The incorporation of m^6^ATP into RNA by pol η in the presence of Mg^2+^.** DNA-templated 1 nt RNA gap substrate and RNA open-template substrate (50 nM) were incubated with increasing concentrations of pol η (25–50 nM) at 37^◦^C for 60 min in the presence of 500 μM ATP or m^6^ATP and 5 mM Mg^2+^. Panel (**A**) represents the pol η RNA synthesis on the 1 nt RNA gap substrate and the quantification of the RNA synthesis products. Panel (**B**) represents the pol η RNA synthesis products on the open RNA template substrate. Lanes 1 and 4 represent substrate only. Lanes 2–3 represent pol η ATP incorporation on the gap (panel **A**) or open-templated RNA substrate. Lanes 5–6 represent pol η m^6^ATP incorporation on gap or open-templated RNA substrate (panel **B**). The synthesized RNA products are indicated by arrows on the left side of the gel images. The percentage of the pol η RNA synthesis products was calculated and illustrated in the bar charts below the gel images. All experiments were performed at least in triplicate.

**Figure 3:**
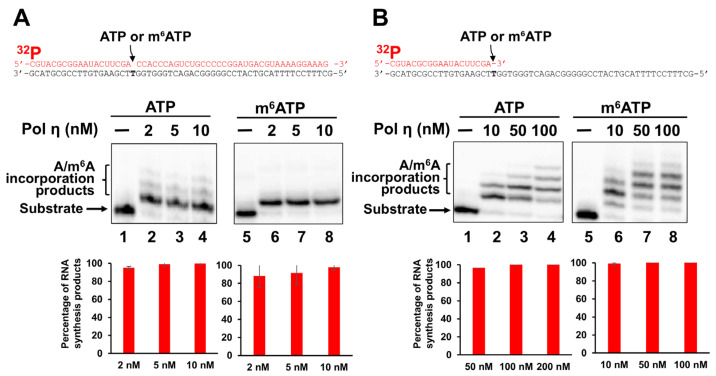
**The incorporation of ATP and m^6^ATP into RNA by pol η in the presence of Mn^2+^.** DNA-templated 1 nt RNA gapped and open-template substrates (50 nM) were incubated with increasing concentrations of pol η (2–10 nM for the RNA gap substrate and 10–100 nM for the RNA open-template substrate) at 37 °C for 60 min in the presence of 500 μM ATP or m^6^ATP and 1 mM Mn^2+^. Panel (**A**) represents the results of pol η RNA incorporation of ATP and m^6^ATP on the 1 nt RNA-gap substrate. Panel (**B**) illustrates the results of pol η incorporation of ATP and m^6^ATP on the RNA open-template substrate. The percentage of RNA synthesis products is illustrated in the bar charts below the gel images. Lanes 1 and 5 represent substrate only. Lanes 2–4 represent pol η ATP incorporation on gap or open-templated RNA substrate. Lanes 6–8 represent m^6^ATP incorporation by pol η on gap or open-templated RNA substrate. The synthesized RNA products are indicated by arrows on the left side of the gel images. All experiments were performed at least in triplicate.

We next asked if pol η can also incorporate m^6^ATP into RNA. As a translesion DNA synthesis polymerase, pol η is known for its structural flexibility and tolerance of diverse nucleotides and DNA damage. Thus, we reasoned that pol η exhibits the ability to incorporate m^6^ATP in the presence of Mg^2+^. To test this, we examined the pol η incorporation of m^6^ATP on the 1 nt RNA gap and open-template RNA substrates in the presence of Mg^2+^ and Mn^2+^ and compared the incorporation activity with that of ATP incorporation (Figure 2). The results indicated that pol η at 25–50 nM efficiently incorporated ATP and m^6^ATP into both the 1 nt gap (Figure 2A, lanes 2–3 and 5–6) and open-template RNA substrates (Figure 2B, lanes 2–3 and 5–6) in the presence of Mg^2+^. Moreover, with both the RNA substrates, pol η extended m^6^A by incorporating additional m^6^A, creating a small amount of m^6^A:dG base mispairing product (Figure 2A, 2B, lanes 5–6). In the presence of Mn^2+^, pol η incorporated ATP and m^6^ATP in both gapped and open-template RNA efficiently (Figure 3). Surprisingly, on the RNA gapped substrate, pol η incorporation of ATP but not m^6^ATP also led to a small amount of A:dG mismatch (Figure 3A, compare lanes 2–4 with lanes 6–8). On the RNA open-template substrate, pol η generated multiple nucleotide misincorporation products with both ATP and m^6^ATP (Figure 3B, lanes 2–4 and 6–8), indicating a significantly reduced fidelity of ATP and m^6^ATP incorporation.

### 2.3. The Catalytic Efficiency of m^6^ATP Incorporation by Pol β and Pol η

**Figure 4:**
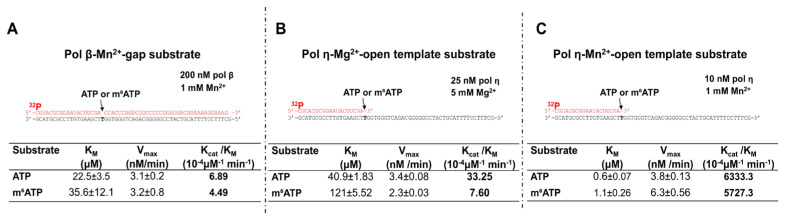
**Steady-state kinetics of ATP and m^6^ATP incorporation by pol β and pol η in the presence of Mn^2+^ and Mg^2+^.** The steady-state kinetics of pol β incorporation of ATP and m^6^ATP in the presence of 1 mM Mn^2+^ (**A**) and pol η incorporation of ATP and m^6^ATP in the presence of 5 mM Mg^2+^ (**B**) and 1 mM Mn^2+^ (**C**) were measured with various concentrations of ATP or m^6^ATP using the 1 nt RNA gap substrate (pol β) or RNA open-template substrate (pol η). The experiments were performed at least in triplicate.

Using steady-state kinetics, we further compared the efficiency of pol β and pol η incorporation of m^6^ATP with that of ATP in the presence of Mg^2+^ and Mn^2+^ (Figure 4). Given the fact that pol β and pol η preferential substrates are 1 nt gapped and open-template substrates, respectively, we performed the steady-state kinetics studies on the RNA synthesis by DNA polymerases. The results showed that pol β exhibited similar catalytic efficiency (*k*_cat_/K_m_) in inserting ATP and m^6^ATP, shown as 6.89 × 10^−4^ μM^−1^ min^−1^ for ATP and 4.49 × 10^−4^ μM^−1^ min^−1^ for m^6^ATP in the presence of Mn^2+^ (Figure 4A). In contrast, in the presence of 5 mM Mg^2+^, the efficiency of pol η incorporation of ATP on the RNA open template exhibited more than four-fold higher catalytic efficiency than that of m^6^ATP (Figure 4B). However, in the presence of 1 mM Mn^2+^, the catalytic efficiency of ATP and m^6^ATP incorporation by pol η was similar, with 6333.3 × 10^−4^ μM^−1^ min^−1^ for ATP incorporation and 5727.3 × 10^−4^ μM^−1^ min^−1^ for m^6^ATP incorporation. The results indicated that the presence of Mn^2+^ significantly stimulated the catalysis of m^6^ATP incorporation by pol η. The catalytic efficiency of pol η m^6^ATP incorporation was increased by nearly 200-fold by Mn^2+^ (compare the *k*_cat_/K_m_ for ATP and m^6^ATP in panel B with *k*_cat_/K_m_ of the nucleotides in panel C). The steady-state kinetics results revealed that pol η inserted m^6^ATP into the RNA open-template substrate with high efficiency, indicating that m^6^ATP was efficiently incorporated by pol η in the presence of Mg^2+^ and Mn^2+^.

### 2.4. The Structural Basis of m^6^ATP RNA Incorporation by Pol β and Pol η in the Presence of Mg^2+^ and Mn^2+^

**Figure 5:**
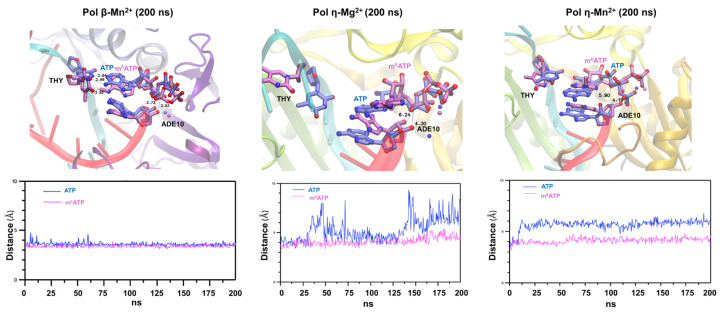
**Structural basis of the incorporation of m^6^ATP by pol β and pol η.** MD experiments were performed with the pol β-1 nt RNA gap-ATP or m^6^ATP ternary complexes in the presence of 10 mM Mn^2+^ and pol η-RNA open-template-ATP or m^6^ATP ternary complexes in the presence of 50 mM Mg^2+^ (the panel in the middle) or in the presence of 10 mM Mn^2+^ (the panel on the right). The MD experiments were performed for 200 ns. The dynamic changes in the distance between the 3′-hydroxyl group of the 3′-terminus primer and α-phosphate of ATP or m^6^ATP were determined and illustrated as RMSD (Å) in the graphs shown below the enzyme–substrate ternary complex structures.

To further reveal the structural basis of the incorporation of m^6^ATP into RNA by pol β and pol η in the presence of Mn^2+^ or Mg^2+^, we conducted an AlphaFold3-assisted molecular dynamics (MD) simulation of m^6^ATP and ATP incorporation by the DNA polymerases using pol β (PDB ID 5TBB) [35] and pol η crystal structures (PDB ID 4J9N) [36], with the DNA sequences of the substrates replaced with the DNA and RNA sequences used in this study. We superimposed the structures of enzyme–substrate ternary complexes with m^6^ATP and ATP for both pol β and pol η to elucidate the molecular mechanisms underlying the different nucleotide incorporation efficiency by pol β and pol η in the presence of Mn^2+^ or Mg^2+^ (Figure 5). The results showed that pol β adopted a different conformation with m^6^ATP from that with ATP in the DNA-RNA-pol β-m^6^A/ATP ternary complex (Figure 5, the left panel). In the pol β-ATP-RNA substrate-Mn^2+^ ternary complex, ATP exhibited its base pair with the template T (Figure 5, the left panel). Surprisingly, m^6^ATP failed to base pair with the template T. Instead, it employed the purine ring to interact with the part of the purine ring of the adenosine at the 3′-terminus of the upstream primer, suggesting a base-stacking-like interaction (Figure 5, the left panel). This led to the almost identical distance between the 3′-OH group and α phosphate of 3.53 Å (ATP) and 3.75 Å (m^6^ATP). The RMSD analysis showed that the distance was stabilized between 3.5 Å and 4.5 Å (Figure 5, the left panel, the RMSD graph below the structure). On the other hand, in the pol η-substrate-ATP complex in the presence of Mg^2+^, ATP formed a hydrogen bond with the template T, resulting in 6.24 Å between the 3′-OH group of the RNA primer and the α phosphate of ATP (Figure 5, the panel in the middle). However, similar to the pol β-m^6^ATP ternary complex, m^6^ATP also failed to base pair with the template T in the pol η-m^6^ATP ternary complex. Instead, it exhibited a partial base-stacking interaction with the purine ring of the 3′-terminus A of the upstream primer. Consequently, this altered the distance between the 3′-OH group and the α-phosphate of m^6^ATP to 4.30 Å (Figure 5, the panel in the middle). The RMSD analysis showed that the distance between the 3′-OH group and the α-phosphate of ATP dynamically changed between 3.5 Å and 9 Å, suggesting an active molecular collision. However, the distance for m^6^ATP was stabilized within the range of 3.5 Å–5.5 Å (Figure 5, the graph below the panel in the middle). Interestingly, in the pol η-substrate-Mn^2+^ ternary complex, ATP failed to base pair with the template T, thereby increasing the distance to 5.90Å for the 3′-OH group and the α-phosphate of ATP (Figure 5, the panel on the right). However, the polymerase employed the base-stacking interaction of m^6^ATP with the purine ring of the 3′-terminus A, thereby shortening the distance of the 3′-OH group and the α-phosphate of m^6^ATP to 4.19Å (Figure 5, the panel on the right). The results were consistent with the RMSD results, showing that the distance for the 3′-OH group of the 3′-terminus nucleotide and the α-phosphate of ATP and m^6^ATP was stabilized at 6Å and 4Å, respectively (Figure 5, the right panel and the graph below the structure). The results further indicated that both pol β and pol η employed the same base-stacking-like strategy to sustain the distance between the 3′-OH and α-phosphate of m^6^ATP for the catalysis of m^6^ATP incorporation in the presence of Mn^2+^ and Mg^2+^.

### 2.5. A Significant Amount of m^6^ATP Is Detected in Human Normal and Cancer Cells and Is Associated with Cellular Oxidative Stress

**Figure 6:**
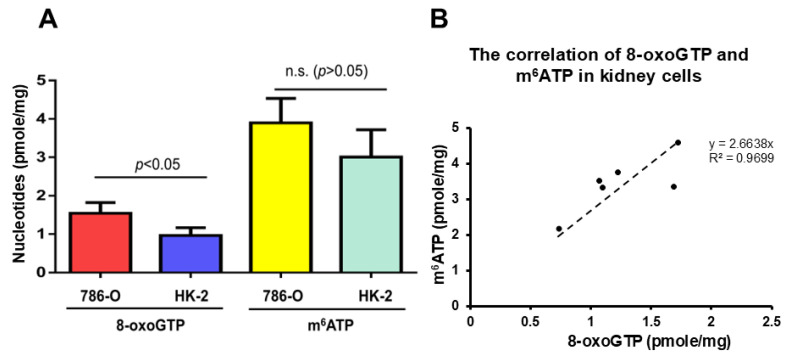
**The level of m^6^ATP and its association with the oxidative stress biomarker 8-oxoGTP in kidney cells.** The m^6^ATP and 8-oxoGTP levels in kidney cancer cells (786-O) and normal kidney cells (HK-2) were measured using liquid chromatography–multiple reaction monitoring mass spectrometry analysis, as described in the Materials and Methods. (**A**) The levels of 8-oxoGTP and m^6^ATP (pmole/mg protein) in 786-O and HK-2 cells were illustrated using a bar chart. (**B**) The correlation between the levels of m^6^ATP and 8-oxoGTP in 786-O and HK2 cells was created by plotting the level of m^6^ATP (Y-axis) and 8-oxoGTP (X-axis) using a linear regression. The results were obtained from three biological replicates of 786-O and HK-2 cells.

To determine if there exists m^6^ATP in the cellular nucleotide pool and if there is an association between the cellular m^6^ATP level and oxidative stress and its-induced RNA gaps, we detected the levels of m^6^ATP and 8-oxoGTP, the most frequently oxidized nucleotides in the nucleotide pool in human cells, which are often used as biomarkers of cellular oxidative stress. We used LC-MRM/MS to measure the cellular m^6^ATP and 8-oxoGTP levels in normal human kidney cells (HK-2) and kidney cancer cells (786-O). We found that m^6^ATP and 8-oxoGTP existed in the cells with 3 pmole/mg protein of m^6^ATP and 0.9 pmole/mg protein of 8-oxoGTP in normal kidney cells, HK-2, along with 3.9 pmole/mg protein of m^6^ATP and 1.5 pmole/mg protein of 8-oxoGTP in kidney cancer cells, 786-O (Figure 6A). Moreover, the results showed that m^6^ATP is 2.6–3-fold higher than 8-oxoGTP in the kidney cancer cells and normal kidney cells, indicating the existence of m^6^ATP in the nucleotide pool. Surprisingly, the results further revealed that the m^6^ATP and 8-oxoGTP levels in the kidney cancer 786-O cells were 1.3-fold (*p* < 0.05) and 1.7-fold (n.s., *p* > 0.05) higher than those in normal kidney cells, respectively, suggesting that there was a correlation between the cellular levels of m^6^ATP and 8-oxoGTP. Indeed, we found that a linear correlation between the levels of m^6^ATP and 8-oxoGTP in kidney cells was established, with the cellular m^6^ATP increasing with increased level of 8-oxoGTP (Figure 6B). Collectively, these results indicate that m^6^ATP can exist in the cellular nucleotide pool at a significant level, suggesting that it is elevated along with cellular oxidative stress and its resulting DNA and RNA damage.

## 3. Discussion

**Figure 7:**
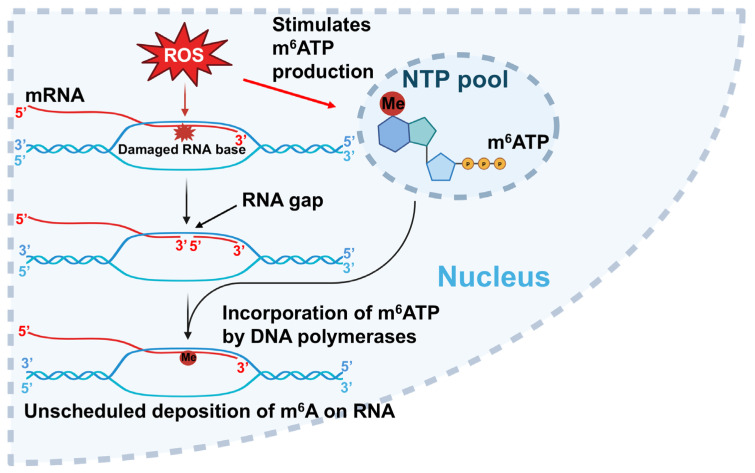
**Unscheduled m^6^A deposition through m^6^ATP incorporation by DNA polymerases induced by oxidative stress.** Cellular oxidative stress stimulates the production of m^6^ATP in the nucleotide pool and induces RNA base damage, such as RNA gaps on an RNA-DNA hybrid during gene transcription. DNA polymerases such as pol β and pol η can then make use of the RNA gaps and take m^6^ATP from the ribonucleotide pool to incorporate m^6^ATP into the RNA strand through their RNA gap-filling synthesis. Subsequently, this results in the unscheduled m^6^A deposition into RNA through the maintenance of RNA integrity by DNA repair polymerases.

In this study, we provided the first evidence that the repair and translesion DNA polymerases pol β and pol η can incorporate m^6^ATP into RNA on the 1 nt RNA gap and RNA open-template substrates with comparable efficiency for their ATP incorporation (Figure 1, Figure 2, Figure 3 and Figure 4). Using MD, we further demonstrated that pol β and pol η incorporated m^6^ATP by employing a unique base-stacking-like mechanism with a part of the purine ring of the 3′-terminus adenosine of the upstream RNA primer in the presence of Mn^2+^ and Mg^2+^. The unique mechanism governed the distance between the 3′-OH group and the α-phosphate of m^6^ATP and its dynamic change, mediating the efficiency of m^6^ATP incorporation (Figure 5). Furthermore, we detected a significant amount of m^6^ATP in normal and cancer kidney cells. We showed that the cellular level of m^6^ATP increased with the increase in the amount of the oxidative stress biomarker 8-oxoGTP (Figure 6A,B). Our results supported the hypothetical model in which cellular oxidative stress from ROS can simultaneously stimulate the production of m^6^ATP in the cellular nucleotide pool and induce RNA damage, such as RNA gaps. Subsequently, RNA gaps are recognized by repairing DNA polymerases, such as pol η and pol β. The DNA polymerases then incorporate m^6^ATP into RNA through their RNA gap-filling synthesis activity on an RNA-DNA hybrid, leading to the unscheduled m^6^A deposition on RNA (Figure 7). There may be a scenario during which, after m^6^A is incorporated, the RNA strand is displaced by the reannealing of the non-template DNA to the template DNA strand, forcing the RNA strand to dissociate from the template. In this case, the RNA strand may be subject to RNA degradation, generating m^6^AMP that can be converted back into m^6^ATP in the nucleotide pool and reused for m^6^ATP RNA incorporation.

Here, we revealed a potential alternative mechanism for m^6^A RNA deposition that is independent of the RNA methyltransferase, METLL3/METTL14, but through m^6^ATP incorporation via oxidative RNA damage-induced RNA gaps by repairing DNA polymerases. We named the alternate pathway as an unscheduled m^6^A RNA deposition. Our study suggests that the unscheduled m^6^A deposition through RNA damage can potentially lead to the disruption of m^6^A RNA depositions that are usually regulated by m^6^A writers such as METTL3/METTL14, thereby altering cellular functions. Moreover, we found that incorporation of m^6^A by pol β and pol η also resulted in nucleotide misincorporation through its extension, especially on the RNA open-template substrate, which represents a large RNA gap on RNA-DNA hybrids (Figure 1B, lanes 6–8, Figure 2A, 2B, lanes 5–6, and Figure 3B, lanes 6–8). This can potentially reduce RNA integrity, protein–RNA interactions, and m^6^A-regulated cellular functions. Our study further suggests crosstalk between RNA modifications and DNA repair polymerase, implicating the interplay of RNA integrity, cellular oxidative stress, and epitranscriptional regulation on R-loops during gene transcription and DNA repair. Our findings will also provide new insights into an alternate mechanism of m^6^A RNA deposition, contributing to a broader understanding of the diversified functions of repairing DNA polymerases that bridge the crosstalk between the genome and epitranscriptome.

Our results suggest that repairing DNA polymerases can make use of RNA base damage, such as AP-site-induced RNA gaps, to incorporate m^6^ATP into RNA on RNA-DNA hybrids. It is possible that the incorporated m^6^A in RNA can be sustained in RNA via its extension by DNA polymerases (Figure 1B, Figure 2, and Figure 3), leading to an RNA nick that is subsequently sealed by an RNA ligase, as recently reported in [37]. Since AP sites are the most abundant form of RNA base damage that can arise from cellular spontaneous depurination/depyrimidination or exposure to endogenous and exogenous genotoxicants [38], this may increase the probability of m^6^ATP being incorporated into RNA via RNA damage. Recent studies have implicated the effects of RNA damage and repair on genome stability via RNA-DNA hybrids on R-loops, and several critical reviews have highlighted the importance of RNA damage and repair in modulating cellular gene expression and function [39,40,41]. Our findings further suggest that repairing DNA polymerases can mediate the crosstalk between the epitranscriptome and genome by playing a dual role in incorporating m^6^ATP and performing RNA-guided DNA gap-filling synthesis to repair DNA base damage [42] on RNA-DNA hybrids. Consequently, this may further allow DNA repair polymerases to coordinate their activities for DNA repair and m^6^A incorporation, thereby modulating epitranscriptomic modifications and maintaining genome integrity.

Structurally, the presence of the methyl group at the N^6^ position of m^6^ATP may cause steric hindrance to disrupt the base-pairing of the nucleotide with the template T. This may further alter the interaction of pol β and pol η with the DNA-RNA hybrid substrates, reducing the fidelity and efficiency of the DNA polymerases. Using AlphaFold3-assisted MD, we found that to adapt to the steric hindrance from the methyl group of m^6^ATP, pol β and pol η evolved a unique base-stacking or base-stacking-like mechanism by employing the purine ring of m^6^ATP to base stack with the part of the purine ring of the 3′-terminus adenosine of the upstream RNA primer (Figure 5). Consequently, this resulted in the stabilized distance between the 3′-OH group and the α-phosphate of m^6^ATP ranging between 3.5 and 4Å (Figure 5), thereby ensuring the success of the nucleophilic attack initiated by the 3′-OH group of the upstream primer and the catalysis of m^6^ATP incorporation. Furthermore, we found that in the pol β-RNA-DNA hybrid-ATP ternary complex in the presence of Mn^2+^, the distance only exhibited a small dynamic change ranging from 3.5 to 4.5Å during 6.25–62.5 ns as compared to the stabilized distance in the ternary complex with m^6^ATP (Figure 5, the panel on the left), leading to the similar catalytic efficiency of ATP and m^6^ATP incorporation by pol β (Figure 4A). Interestingly, for pol η, in the presence of Mg^2+^, although the distance between the 3′-OH group and α-phosphate of ATP underwent a large range of dynamic changes, ranging from 3.5Å to 9Å as compared with the change from 4 to 5 Å for m^6^ATP (Figure 5, the panel in the middle), the DNA polymerase still achieved more than four-fold higher efficiency in incorporating ATP than m^6^ATP in the presence of Mg^2+^ (Figure 4B). In the presence of Mn^2+^, pol η maintained the distance change for ATP from 4.5Å to 6Å and 4Å to 4.5Å for m^6^ATP during 200 ns, leading to a higher efficiency of ATP incorporation than m^6^ATP. These results suggest that the large dynamic change in the distance between the 3′-OH group and α-phosphate of ATP and m^6^ATP, rather than the distance per se, plays a crucial role in mediating the catalysis of the nucleotide incorporation into RNA. The results further demonstrate that the differences in the dynamic distance change between the 3′-OH group and the α-phosphate of the incoming nucleotides in pol β and pol η determined the efficiency of the molecular collision of the atoms, thereby governing the difference in the catalytic efficiency of ATP and m^6^ATP incorporation.

In this study, we detected a significant amount of m^6^ATP in normal kidney and kidney cancer cells (Figure 6A). We further identified a positive correlation between the levels of m^6^A and the oxidative stress biomarker 8-oxoGTP (Figure 6B), suggesting that the cellular m^6^ATP level can also serve as an effective response to oxidative stress. Furthermore, since oxidative stress that is signified by the massive production of cellular ROS can simultaneously damage DNA, RNA, and nucleotides triphosphate in the nucleotide pool, we suggest that an unscheduled m^6^A deposition can be mediated via m^6^ATP incorporation into RNA gaps by DNA polymerases upon RNA damage on RNA-DNA hybrids on R-loops induced by oxidative stress (Figure 7). Moreover, our results show that the level of m^6^ATP is 3- and 2.6-fold of 8-oxoGTP in normal kidney cells and kidney cancer cells (Figure 6A), suggesting that the level of m^6^ATP in the cellular nucleotide pool is significant and plays a critical role in modulating RNA modifications and m^6^A deposition in an RNA methyltransferase-independent mechanism. Since it was reported that m^6^A in DNA can be generated by incorporating m^6^dATP [43], our results suggest that m^6^ATP in the nucleotide pool can also be used as the precursor to be reduced into m^6^dATP for generating ^6^mA in DNA, leading to the maintenance of genome stability, as reported recently [44]. Since Musheev et al. have shown that m^6^AMP can be converted into m^6^ADP, which is then reduced to m^6^dADP by ribonucleotide diphosphate reductase, which is subsequently, phosphorylated into m^6^dATP, leading to the incorporation of ^6^mATP into DNA by DNA polymerases [43], it is possible that cellular m^6^ATP in the ribonucleotide pool can also be directly regenerated from m^6^AMP that results from the degradation of m^6^A-modified RNA. m^6^AMP can then be phosphorylated into m^6^ATP by a nucleotide kinase and recycled back into the nucleotide pool. Thus, our study opens a new avenue for revealing the novel role of m^6^A in mediating the interplay between epitranscriptomic and genomic stability. It should be noted that our results show that kidney cancer cells exhibited higher m^6^ATP and 8-oxoGTP levels than normal kidney cells, without a statistically significant difference (*p* > 0.05) for m^6^ATP but with a statistically significant difference for 8-oxoGTP (*p* < 0.05) (Figure 6A). This further indicates that more biological replicates will be needed to determine the statistical significance of the difference in m^6^ATP between normal and cancer kidney cells.

It has been reported that in mammalian cells, the intracellular concentrations are approximately ATP 3152 ± 1698 µM, GTP 468 ± 224 µM, UTP 567 ± 460 µM, and CTP, 278 ± 242 µM [45]. In contrast, the concentrations of dNTPs exhibit different ranges: dATP 24 ± 22 µM, dGTP 5.2 ± 4.5 µM, dCTP 29 ± 19 µM, and dTTP 37 ± 30 µM [45]. Based on the fact that a single mammalian kidney cell has a volume of 96 μm^3^ per cell (use rat kidney duct cells as an example), as reported in [46], according to our results, we estimated that the concentrations of m^6^ATP in 786-O cells and normal kidney HK-2 cells were 33.8 ± 4.9 nM and 26.1 ± 5.5 nM, respectively. Thus, the m^6^ATP concentration in the human kidney cells was about 1000-fold lower than the level of dATP in cells. Since Musheev et al. have found that m^6^dATP at a lower concentration than m^6^ATP can be incorporated into the genomic DNA by DNA polymerases in cells [43], our results suggest that it is possible that m^6^ATP at a relatively higher concentration than m^6^dATP in cells can also be incorporated into RNA on RNA-DNA hybrids of R-loops.

In cells, RNA gaps with a DNA template can be induced on R-loops. This can result from RNA base damage such as RNA abasic sites that are generated by cellular oxidative stress and RNA base alkylation [38]. Subsequently, RNA abasic sites on RNA-DNA hybrids can be cleaved by human AP endonuclease 1 (APE1) at the 5′-end of the abasic sites, leading to the formation of RNA gaps on RNA-DNA hybrids [47]. It is possible that RNA gaps also serve as the substrates for RNase H1 on R-loops, leading to coordination between RNase H1 and DNA repair polymerase-mediated m^6^ATP incorporation. However, it is unlikely that RNA gaps are used as the substrate of RNA polymerases. This is because RNA polymerases recognize the template DNA as the substrate and move along with the template DNA to perform RNA synthesis. Thus, it is unlikely that RNA polymerases can readily move back to bind RNA gaps on RNA-DNA hybrids. Here, we discovered an alternative role of repairing DNA polymerases with their RNA m^6^ATP incorporation activity due to their substrate plasticity. Studies on understanding the coordination among DNA repair polymerases and RNA processing enzymes in modulating m^6^ATP incorporation shall be conducted in the future.

It was reported that the concentration of Mn^2+^ in human cells is typically low and that the physiological concentration of Mn^2+^ in human HeLa cells is 1.14 ± 0.15 μM [48]. This concentration is significantly lower than the concentration used in our experiments (1 mM), which can represent an Mn^2+^ genotoxic level. Although we did not detect any m^6^ATP incorporation by the DNA polymerases at the physiological concentration of Mn^2+^, it remains possible that the incorporation of m^6^ATP by pol β and pol η in cells can be induced at the physiological concentration of Mn^2+^ of 1.14 μM by cooperating with their cofactors. The importance of m^6^ATP incorporation by DNA repair polymerases in the presence of their cofactors under physiological and pathological concentrations of Mn^2+^ needs to be elucidated in the future.

Our MD results showed that the methyl group on m^6^ATP disrupted the standard Watson–Crick base pairing between m^6^ATP and the template T in the catalytic center of the DNA polymerases by forcing the purine ring m^6^ATP to turn away from the template T, leading to reduced efficiency of m^6^ATP incorporation. Simultaneously, the methyl group promoted the base-stacking interaction of m^6^ATP with the upstream primer 3′-terminus A through hydrophobic and van der Waals effects, thereby sustaining the distance between the 3′-OH of the upstream primer and the α-phosphate of m^6^ATP needed for catalysis of the nucleotide incorporation. Our results further indicate that the methyl group at the 6-position of m^6^ATP forced the purine ring of the nucleotide to form base-stacking interactions with the part of the aromatic ring of 3-terminus A of the upstream primer (Figure 5) via π–π interactions, which stabilizes the orientation of the 3′-OH group of the upstream RNA primer. This further suggests that the upstream primer 3′-terminus nucleotide does not strictly need to be adenine to form its base-stacking interaction with the purine ring of m^6^ATP. The effects of various types of ribonucleotides at the 3′-terminus on the base-stacking interaction with m^6^ATP warrant a study in the future.

However, it should be noted that our study has several limitations. First, while our results provide new insights into the potential mechanism of unscheduled m^6^A deposition mediated by repairing DNA polymerases, which was associated with oxidative stress, its physiological functions on epitranscriptomic regulation and genome stability remain to be elucidated in vivo using animal models or advanced cellular models in the future. In addition, we could not exclude the possibility that the association between m^6^ATP and 8-oxoGTP may be affected by some potential cofounding factors in cells. The causal correlation between oxidative-stress-induced 8-oxoGTP and m^6^ATP and their effects on cellular functions and disease progression needs to be further determined. Second, the conditions employed in our in vitro RNA-DNA hybrid-based approaches may not fully reflect those in complex biological systems. However, our results suggest the possibility of m^6^ATP RNA incorporation by DNA repair polymerases in cells. This is supported by the fact that pol β and pol η are well known to adapt to the noncanonical substrates to tolerate and incorporate oxidized, damaged, and other types of modified nucleotides [49,50]. Moreover, our results show that pol η exhibited efficient incorporation of m^6^ATP in RNA at a physiological concentration of Mg^2+^, suggesting the potential role of the DNA polymerase in incorporating modified ribonucleotide in RNA in cells. Also, it is possible that in cells, the repair DNA polymerases may enrich m^6^ATP and Mg^2+^ or Mn^2+^ at the enzyme catalytic center to a level comparable to that used in our experiments, thereby fulfilling their efficient catalysis for m^6^ATP incorporation in RNA. The mechanisms of m^6^ATP RNA incorporation by repairing DNA polymerases via RNA damage need to be further elucidated in vivo. Third, in our study, the levels of m^6^ATP and 8-oxoGTP were detected only in limited types of cells, the normal and cancer kidney cells. This may limit the application of our conclusions to a broader range of cell types, including healthy and diseased cells. In addition, our results have a limitation in revealing the relative abundance of m^6^ATP and 8-oxoGTP in the cellular nucleotide pool due to the lack of information on the total amount of ATP and GTP in the kidney cell lines. Future studies should also determine the percentage of chemically modified ribonucleotides among the total amount of all four types of ribonucleotides in the cellular nucleotide pool to elucidate the roles of the modified nucleotides in modulating cellular functions. Furthermore, future studies on addressing all these limitations will reveal the biological significance and impact of the unscheduled m^6^A deposition, opening a new avenue for understanding the crosstalk between epitranscriptome and genome via m^6^A incorporation and DNA repair mediated by DNA polymerases on R-loops.

## 4. Materials and Methods

### 4.1. Materials

ATP (100 mM) and m^6^ATP (100 mM) were purchased from Fisher Scientific (Waltham, PA, USA) and TriLink BioTechnologies (San Diego, CA, USA), respectively. The radionucleotide, ^32^P-ATP (6000 µCi/mmol), was purchased from Revvity Inc. (Waltham, MA, USA). DNA and RNA oligonucleotides were synthesized by Eurofins Genomics (Louisville, KY, USA). Micro Bio-Spin 6 chromatography columns were from Bio-Rad Laboratories (Hercules, CA, USA). Human pol β protein was expressed and purified as described previously [51]. Purified human pol η protein was a generous gift from Dr. Wei Yang from the National Institute of Diabetes and Digestive and Kidney Diseases (NIDDK)/National Institutes of Health (NIH) [36]. BER reaction buffer was made with 50 mM Tris-HCl, pH 7.5, 50 mM KCl, 0.1 mM EDTA, 0.1 mg/mL bovine serum albumin (BSA), and 0.01% Nonidet P-40 (NP-40). Moreover, 2× stopping buffer was made of 95% deionized formamide and 10 mM EDTA, 0.25% bromophenol blue (Sigma-Aldrich, St. Louis, MO, USA), and 0.25% (*w/v*) xylene cyanol FF (Sigma-Aldrich, St. Louis, MO, USA).

### 4.2. RNA-DNA Hybrid Oligonucleotide Substrates

An RNA-DNA hybrid substrate containing a 1 nt RNA gap was designed to mimic an RNA damage intermediate to test the 1 nt RNA gap-filling synthesis. An open-template RNA substrate was designed to mimic a large RNA gap generated from RNA damage. The 1 nt gap substrate was constructed by annealing the 5’-end ^32^P-ATP radiolabeled 19 nt upstream RNA primer (5′-CGUACGCGGAAUACUUCGA-3′) and 36 nt downstream RNA primer (5′-CCACCCAGUCUGCCCCCGGAUGACGUAAAAGGAAAG -3′) with the 57 nt DNA template containing a dT base positioned opposite to the 1 nt gap (5′-GCTTTCCTTTTACGTCATCCGGGGGCAGACTGGGTGGTTCGAAGTGTTCCGCGTACG-3′. The 1 nt gap substrate was assembled by annealing the upstream and downstream RNA primers with the template strand at a molar ratio of 1:3:3. An open-template RNA-DNA hybrid substrate was constructed by annealing the 5’-end ^32^P-ATP-radiolabeled 19 nt upstream RNA primer with the 56 nt DNA template at a molar ratio at 1:3. The RNA and DNA oligonucleotides were annealed in 1× annealing buffer containing 10 mM Tris pH 7.5, 100 mM NaCl, and 1 mM EDTA. The substrate annealing mixture was then subjected to 95 °C for 5 min and slowly cooled down to room temperature at 25 °C.

### 4.3. Determination of RNA Synthesis Activity by DNA Polymerases

The DNA-templated RNA synthesis activities of pol β and pol η were measured by incubating 50 nM RNA-DNA hybrid substrates with increasing concentrations of the DNA polymerases in the presence of 500 μM NTPs at 37 °C for 60 min in the reaction mixture (20 µL containing 5 mM of Mg^2+^ or 1 mM of Mn^2+^ in the BER reaction buffer with 50 mM Tris-HCl, pH 7.5, 0.1 mM EDTA, 50 mM KCl, 0.01% NP-40 and 0.1 mg/mL BSA). Under these conditions, DNA polymerases were able to achieve high ribonucleotide incorporation efficiency, as reported in previous studies [52,53]. The reactions were terminated by adding a 2× stop buffer (10 mM EDTA, 95% deionized formamide, and 0.25% (*w/v*) each of bromophenol blue and xylene cyanol FF). Samples were then denatured at 95 °C for 5 min and subjected to 15% urea-denaturing polyacrylamide gel electrophoresis. The substrates and products were detected using a Pharos FX Plus PhosphorImager (Bio-Rad Laboratories, Hercules, CA, USA). All the experiments were repeated independently three times.

### 4.4. Determination of the Efficiency of ATP and m^6^ATP Incorporation in RNA by Pol β and Pol η Using Steady-State Kinetics

The efficiency of ATP and m^6^ATP incorporation in RNA by pol β and pol η was determined using the steady-state enzyme kinetics of RNA synthesis at a fixed concentration of the DNA polymerases, RNA-DNA hybrid substrate, and metal cofactor (Mg^2+^ or Mn^2+^) with increasing concentrations of ATP or m^6^ATP substrates. The steady-state kinetics of ATP and m^6^ATP incorporation by pol β was determined using 50 nM of 1 nt gap substrate, 200 nM of pol β, and 1 mM of Mn^2+^ with increasing concentrations of ATP and m^6^ATP (10–100 μM). The steady-state kinetics of RNA synthesis by pol η was determined using 50 nM of open-template RNA substrate, and either 25 nM of pol η, 5 mM of Mg^2+^ with increasing concentrations of ATP and m^6^ATP (10–1000 μM) or 10 nM of pol η, 1 mM of Mn^2+^ with increasing concentrations of ATP and m^6^ATP (0.5–5 μM). The enzymes and substrates were incubated at 37 °C for different time intervals (0 to 15 min for pol β and pol η-Mg^2+^, 0–10 min for pol η-Mn^2+^) in the reaction mixture (20 μL). The reactions were terminated using a 2× stop buffer, and the reaction mixture was heated for 5 min at 95 °C. Substrates and products were separated using a 15% urea-denaturing polyacrylamide gel and detected by the Pharos FX Plus PhosphorImager. The V_max_, K_m_, and *k*_cat_ of the DNA polymerases were calculated using the Enzyme Kinetics Module of the Prism-GraphPad software version 9.0.2. All the experiments were repeated at least three times.

### 4.5. Molecular Dynamics Simulation of the Ternary Complexes of Pol β or Pol η-RNA-DNA Hybrid-NTPs

Protein–RNA-DNA hybrid-NTP ternary complexes were modeled using AlphaFold3 [54] by incorporating the amino acid sequences for human pol β (residues 1–335) or pol η (residues 1–432), along with the 1 nt RNA gap substrate for pol β and RNA open-template substrate for pol η containing the DNA template and up- and downstream primers with the sequences in this study. An ATP molecule and two divalent metal ions, Mg^2+^ or Mn^2+^ were also included in the input structures. The resulting models were structurally aligned to the crystal structures of the pol β ternary complex (PDB: 5TBB) [35] and pol η (PDB: 4J9N) [36,55] for validation and refinement. To generate complexes containing m^6^ATP, the ATP coordinates from the AlphaFold3-generated protein–RNA-DNA ternary complexes were modified using Avogadro2 [56]. A methyl group was added at the N^6^ position of the adenine to yield m^6^ATP, which substituted the ATP molecule in the original models. The pol β and pol η with the substrates were then set up for MD simulations using CHARMM-GUI [57,58,59], with the structures solvated in the TIP3P water model in a cubic box. Ions were added to achieve concentrations of 0.15 M KCl and 50 mM MgCl_2_ for the complex containing Mg^2+^ and 10 mM MnCl_2_ for the complex containing Mn^2+^. The GPU version of NAMD 3.0b2 [60] was used to simulate the systems using Charmm36m force field [61,62]. Simulations began with 10,000 steps of minimization and 250 ps of equilibration at 303 K and 1 atm pressure. The temperature was maintained at 37 °C using Langevin temperature coupling with a damping coefficient of 1/ps. The pressure was kept constant using a Nose–Hoover Langevin piston [63] with a 50-femtoseconds (fs) period and 25 fs decay. The Particle Mesh Ewald method (PME) [64] was used for long-range electrostatic interactions with periodic boundary conditions. All the covalently bonded hydrogen atoms were restrained with the ShakeH algorithm [65]. A 200 ns unconstrained production run was performed for each system using a 2 fs/step. Visual molecular dynamics (VMD) was used to analyze the trajectories.

### 4.6. Cellular m^6^ATP Level Measurement

The levels of m^6^ATP and 8-oxoGTP in human kidney cells (1.2 × 10^6^ cells) (HK-2, ATCC, Manassas, VA, USA) and human kidney cancer cells (1.2 × 10^6^ cells) (786-O, ATCC, Manassas, VA, USA) were determined by liquid chromatography–multiple reaction monitoring mass spectrometry analysis (LC-MRM/MS by Creative Proteomics (Shirley, NY, USA). The LC-MRM/MS platform used by Creative Proteomics (Shirley, NY, USA) employed UPLC–QTRAP 6500+, which was operated in ESI-negative mode with an ammonium acetate/acetonitrile binary gradient. The technique ionized the samples and collected the sample ions in a negative-ion mode. This allowed us to use the retention time to distinguish the methylation group located at different atoms of RNA bases. This approach is commonly used for nucleotide isomer separation and detection, as previously reported [66]. Thus, the method used by Creative Proteomics in our study allowed the differentiation of m^6^ATP from other forms of methylated ATP in cells with an identical *m*/*z* value. In our study, cell pellets from the biological triplicates of two types of cells were used for the experiments. A mixed standard solution containing the synthesized m^6^ATP and 8-oxGTP was used as the internal standard (IS). The standard curves were created via linear regression using the data obtained from these standard solutions. Cell pellets were thawed on ice, resuspended in 15 μL of liquid, and diluted to a final volume of 100 μL by adding 80% acetonitrile. Cells were lysed using an MM 400 mixer mill at 30 Hz for 3 min. Cell lysates were then centrifuged at 21,000× *g* for 10 min to obtain the supernatant. The residual protein pellets were subjected to protein quantification using the standard BCA (bicinchoninic acid) assay. Aliquots (10 μL) of both the cell samples and standard solution were injected into a HILIC column for LC-MRM/MS analysis, which was performed on a Waters UPLC system coupled with a Sciex QTRAP 6500+ mass spectrometer operated in negative-ion detection mode. Chromatographic separation was achieved using binary solvent gradient elution with ammonium acetate buffer and acetonitrile as the mobile phases. The levels of m^6^ATP and 8-oxoGTP in the cell samples were determined by interpolating the standard curve at pmole/mg protein, where “mg protein” refers to the amount of protein per mg protein among the total amount of protein from the total 1.2 × 10^6^ cells of each type of cell. The protein concentrations were measured from the same sample after extraction using a standardized BCA assay.

## 5. Conclusions

In this study, for the first time, we identified a novel mechanism by which m^6^A was deposited into RNA through the incorporation of m^6^ATP by DNA repair and translesion polymerases pol β and pol η through their RNA gap-filling synthesis. We further demonstrated that m^6^ATP was efficiently incorporated into RNA gap and open-template RNA substrates via a unique base-stacking-like mechanism. Further analysis of the cellular m^6^ATP levels showed their positive correlation with the oxidative stress biomarker 8-oxoGTP, suggesting an association of m^6^ATP and its RNA incorporation with cellular oxidative stress. Our findings suggest an interplay among DNA repair, RNA integrity, and genome stability.

## Data Availability

All the data will be deposited into a publicly accessible repository, zenodo: 10.5281/zenodo.16741489.
